# Investigation into the effects of commencing haemodialysis in the critically ill

**DOI:** 10.1186/cc10966

**Published:** 2012-03-20

**Authors:** R Docking, L Moss, M Sim, D Sleeman, J Kinsella

**Affiliations:** 1 University of Glasgow, UK; 2 University of Aberdeen, UK

## Introduction

We aimed to describe haemodynamic changes when haemodialysis is instituted in the critically ill. Three hypotheses are tested: (1) the initial session is associated with cardiovascular instability; (2) the initial session is associated with more cardiovascular instability compared to subsequent sessions; and (3) looking at unstable sessions alone, there will be a greater proportion of potentially harmful changes in the initial sessions compared to subsequent ones.

## Methods

Data were collected for 209 patients, identifying 1,605 dialysis sessions. Analysis was performed on hourly records, classifying sessions as stable/unstable by a cut-off >±20% change in baseline physiology (HR/MAP). Data from 3 hours prior to and 4 hours after dialysis were included, and average and minimum values derived. Three time comparisons were made (pre-HD:during, during HD:post, pre-HD:post). Initial sessions were analysed separately from subsequent sessions to derive two groups. If a session was identified as being unstable, then the nature of instability was examined by recording whether changes crossed defined physiological ranges. The changes seen in unstable sessions could be described as to their effects: being harmful/potentially harmful, or beneficial/potentially beneficial.

## Results

Discarding incomplete data, 181 initial and 1,382 subsequent sessions were analysed. A session was deemed to be stable if there was no significant change (>±20%) in the time-averaged or minimum MAP/HR across time comparisons. By this definition 85/181 initial sessions were unstable (47%, 95% CI SEM 39.8 to 54.2). Therefore Hypothesis 1 is accepted. This compares to 44% of subsequent sessions (95% CI 41.1 to 46.3). Comparing these proportions and their respective CI gives a 95% CI for the standard error of the difference of -4% to 10%. Therefore Hypothesis 2 is rejected. In initial sessions there were 92/1,020 harmful changes. This gives a proportion of 9.0% (95% CI SEM 7.4 to 10.9). In the subsequent sessions there were 712/7,248 harmful changes. This gives a proportion of 9.8% (95% CI SEM 9.1 to 10.5). Comparing the two unpaired proportions gives a difference of -0.08% with a 95% CI of the SE of the difference of -2.5 to +1.2. Hypothesis 3 is rejected. Fisher's exact test gives a result of *P *= 0.68, reinforcing the lack of significant variance.

## Conclusion

Our results reject the claims that using haemodialysis is an inherently unstable choice of therapy. Although proportionally more of the initial sessions are classed as unstable, the majority of MAP and HR changes are beneficial in nature.

